# Characterization of the Complete Mitochondrial Genome of *Pedicularis henryi* and Its Phylogenetic Implications in Lamiales

**DOI:** 10.3390/biology15070586

**Published:** 2026-04-06

**Authors:** Ying Deng, Hong Zhao, Yang Wang, Tian Tian, Zuhong Fan, Fangfang Luo, Ping You

**Affiliations:** School of Ecological Engineering, Guizhou University of Engineering Science, Bijie 551700, China; dy3594142@163.com (Y.D.);

**Keywords:** *Pedicularis henryi*, mitochondrial genome, Orobanchaceae, RNA editing, phylogeny

## Abstract

*Pedicularis henryi* is a hemiparasitic species within Orobanchaceae (Lamiales). In this study, the mitochondrial genome of *P. henryi* was assembled and characterized. The mitogenome is 251,317 bp in length with a GC content of 44.32%, containing 36 protein-coding genes, 24 tRNAs, and three rRNAs. Codon usage analysis revealed a marked preference for A/U-terminated codons. A total of 196 repetitive elements were identified, with interspersed repeats as the most abundant type. We detected 293 C-to-U RNA editing sites across 31 protein-coding genes, predominantly causing non-synonymous substitutions. Eighteen chloroplast-derived fragments totaling 35,894 bp were found, accounting for 15.0% of the mitogenome. Nucleotide diversity analysis among three Pedicularis species showed an average π of 0.0018, with core respiratory genes highly conserved. Synteny analysis revealed extensive structural rearrangement in *P. henryi* compared to *P. chinensis*. This study provides mitochondrial genomic resources for Orobanchaceae and insights into mitogenome evolution in hemiparasitic plants.

## Simple Summary

Understanding how plants adapt to their distinct lifestyles is essential for biodiversity conservation and the management of species interacting with agricultural crops. Hemiparasitic plants, which perform photosynthesis while deriving nutrients from host plants, remain underexplored in terms of their mitochondrial genomes, which are critical for plant growth and evolution. In this study, we assembled and comprehensively characterized the mitochondrial genome of *Pedicularis henryi*, a hemiparasitic flowering plant, and investigated its evolutionary features in comparison with related species. Specifically, we obtained the complete mitochondrial genome sequence, identified its functional genes, repetitive elements, small genetic modifications, and chloroplast-derived DNA fragments. Our results indicate that core energy-production genes are highly conserved across related species, whereas the overall genome architecture has undergone extensive rearrangement compared to its relatives. This work provides essential genetic resources for hemiparasitic plants, deepens our understanding of their evolutionary processes, and offers a foundational reference for plant diversity conservation and agricultural management.

## 1. Introduction

Mitochondria are semi-autonomous organelles responsible for energy production in eukaryotic cells [1]. Since the discovery of mitochondrial DNA in the 1960s [2], research has confirmed that mitochondria possess a complete genetic system, including DNA polymerases, RNA polymerases, and ribosomal components [3,4]. Compared to animal mitochondrial genomes and plant plastid genomes, plant mitochondrial genomes exhibit greater complexity, characterized by larger sizes, frequent structural rearrangements, and extensive incorporation of foreign DNA from chloroplast and nuclear genomes [5,6]. Genome size varies substantially across species, ranging from 66 kb in *Viscum scurruloides* to 11,700 kb in *Larix sibirica* [7,8], primarily due to the accumulation of repetitive sequences and recombination events [9]. Advances in high-throughput sequencing have enabled the assembly of mitochondrial genomes from over 500 plant species, providing valuable resources for evolutionary and functional studies [10,11].

The genus *Pedicularis* (Orobanchaceae) comprises approximately 568 recognized species globally, with China harboring about 350 species, of which 271 are endemic [12,13]. Species within this genus exhibit strong differentiation, complex morphological variation, and diverse habitats due to the dual effects of natural reproductive isolation and rapid radial differentiation, resulting in a rich and diverse genus [14,15]. On the one hand, this diversity provides extremely rich germplasm resources for genetic breeding; on the other hand, it presents great difficulties for taxonomic and systematic studies [16]. Previous genomic investigations have focused primarily on chloroplast genomes, revealing unusual structural features in *Pedicularis* [17,18]. However, despite these advances in understanding plastid genome evolution, the mitochondrial genomes of *Pedicularis* species remain completely unexplored, representing a significant gap in understanding organellar genome evolution within this lineage [19,20].

*Pedicularis henryi*, a hemiparasitic perennial herb of the family Orobanchaceae, is natively distributed across southern China, predominantly inhabiting montane grassy slopes, forest margins and riparian zones at 300–2000 m above sea level [21], and plays critical ecological roles in mediating vegetation succession, soil and water conservation, and local biodiversity maintenance in subtropical montane ecosystems [22]. The genus *Pedicularis* is a hyperdiverse Northern Hemisphere hemiparasitic lineage, renowned for its rapid adaptive radiation, highly specialized floral syndromes and diverse host adaptation strategies, making it a pivotal model system for investigating the evolution of angiosperm parasitic habits, alpine plant speciation dynamics and plant-host ecological interactions. While a small number of mitochondrial genomes have been reported for several *Pedicularis* species, existing genomic resources are far from sufficient to cover the genus’ extraordinary species diversity and complex phylogenetic structure; notably, the complete mitochondrial genome of *P. henryi* remains uncharacterized to date, a critical resource gap that severely limits our understanding of the genetic basis underlying its adaptation to heterogeneous montane environments, its accurate intrageneric phylogenetic placement, and its lineage-specific evolutionary trajectory [23]. Plant mitochondrial genomes serve as indispensable molecular tools for resolving fine-scale plant phylogenetic relationships and dissecting organellar genome evolutionary dynamics in hemiparasitic lineages, thus the assembly and in-depth analysis of the *P. henryi* mitochondrial genome are urgently needed to enrich the genus’ organellar genomic resources and advance related evolutionary research [24]. In this study, we assembled and comprehensively analyzed the complete mitochondrial genome of *P. henryi* using a robust hybrid assembly strategy integrating Illumina short-read and Oxford Nanopore long-read sequencing data [25], systematically characterized its genomic architecture, protein-coding gene repertoire, codon usage bias, putative RNA editing sites, repetitive sequence landscapes and mitochondrial-chloroplast homologous DNA fragments, and further performed comparative genomic and robust phylogenetic analyses to clarify its evolutionary position within *Pedicularis*. This work reports the first complete mitochondrial genome of *P. henryi*, filling the long-standing organellar genomic resource gap for this species, providing an essential foundational reference for future population genetic, phylogenomic and adaptive evolution studies of *Pedicularis*, and advancing our broader understanding of mitochondrial genome evolution in hemiparasitic angiosperms.

## 2. Materials and Methods

### 2.1. Plant Material, DNA Extraction, and Sequencing

*P. henryi* was cultivated under controlled conditions in the greenhouse of Guizhou University of Engineering Science, located at 27°17′34.436″ N, 105°18′46.069″ E. Fresh leaves were harvested from healthy individuals, immediately frozen in liquid nitrogen, and stored at −80 °C until DNA extraction. Total genomic DNA was isolated using a modified CTAB method supplemented with β-mercaptoethanol to minimize oxidation [26]. The purity and concentration of the extracted genomic DNA were assessed with a NanoDrop spectrophotometer and Qubit fluorometer, and its integrity was verified via electrophoresis on a 1.0% agarose gel. High-quality genomic DNA meeting sequencing requirements was subsequently used for short-read and long-read sequencing. A 150 bp paired-end Illumina library was constructed and sequenced on the NovaSeq 6000 platform, generating approximately 10 Gb of clean data; long-read sequencing was performed on the Oxford Nanopore PromethION system, with a corresponding data output of 10 Gb.

### 2.2. Mitochondrial Genome Assembly and Annotation

To reconstruct the complete mitochondrial genome of *P. henryi*, a hybrid assembly strategy combining long and short reads was employed. First, long reads were aligned against a reference set of plant mitochondrial core genes using Minimap2 (v2.1) [27]. Alignments longer than 50 bp were extracted as candidate sequences. Among them, the sequence that covered the largest number of core genes with the highest alignment quality was selected as the seed sequence. This seed was then used as a bait to recruit additional long reads: all original long reads were realigned to the seed with Minimap2, and reads showing an overlap of at least 1 kb and ≥70% identity were iteratively added to extend the seed. Through this iterative procedure, a complete set of long reads belonging to the mitogenome was obtained.

The collected long reads were subsequently corrected using Canu (v2.2) to reduce sequencing errors [28]. Meanwhile, Illumina short reads were aligned to the corrected long-read assembly with Bowtie2 (v2.3.5.1) [29]. Hybrid assembly of both corrected long reads and short reads was then performed with Unicycler (v0.4.8) under default parameters [30], yielding a circular mitochondrial genome with an average sequencing depth of 325×. The assembly graph was visualized in Bandage (v0.8.1) to confirm circularization and to resolve any remaining ambiguities [31]. Final validation was achieved by mapping the original long reads back to the assembled genome using Minimap2 and inspecting coverage uniformity in IGV [32].

Gene annotation was performed through an integrated pipeline. Protein-coding genes (PCGs) were predicted with IPMGA and GeSeq, followed by manual curation in Apollo [33,34]. Transfer RNA genes were identified using tRNAscan-SE (v2.0) [35], and ribosomal RNA genes were detected via BLASTN (v2.15.0) searches against published plant mitochondrial genomes. The annotated mitogenome was graphically represented using OGDRAW (v1.3.1) [36].

### 2.3. Analysis of Codon Usage, RNA Editing Sites, and Repetitive Sequences

Codon usage bias was assessed with CodonW (v1.4.2) based on relative synonymous codon usage (RSCU) values calculated from all PCGs. Putative RNA editing sites in mitochondrial PCGs were predicted using the Deepred-Mt web server, a deep learning tool trained on plant mitochondrial data; only sites with a probability score >0.9 were retained for further analysis [37].

Repetitive elements were characterized using multiple approaches. Simple sequence repeats (SSRs) were detected with MISA (v2.1), applying minimum repeat thresholds of 10, 5, 4, 3, 3, and 3 for mono- to hexanucleotide motifs, respectively [38]. Tandem repeats were identified using Tandem Repeats Finder (v4.09) with default settings [39]. Dispersed repeats (≥30 bp, ≥80% identity) were found by BLASTN self-alignment (E-value ≤ 1 × 10^−5^). All identified repeats were categorized by type and visualized with Circos (v0.69-5) [40].

### 2.4. Chloroplast–Mitochondrial Homology Analysis

Homologous fragments between the mitochondrial and chloroplast genomes were detected via BLASTN searches (E-value ≤ 1 × 10^−5^, identity ≥ 80%, length ≥ 100 bp). Fragments satisfying these criteria were regarded as mitochondrial plastid DNA (MTPT) insertions. The transfer events were visualized using TBtools (v2.309) to illustrate inter-organellar DNA exchanges [41].

### 2.5. Nucleotide Diversity Analysis

Genetic variation among mitochondrial PCGs across the selected Lamiales species was evaluated by calculating nucleotide diversity (π). For each conserved PCG, multiple sequence alignments were generated using MAFFT (v7.427) with default parameters. Pairwise π values were then estimated from the alignments using DnaSP (v5.10) with a sliding window approach (window length: 200 bp; step size: 20 bp) to capture regional variability [42]. The resulting π values were plotted as line graphs to highlight genes or regions exhibiting elevated diversity.

### 2.6. Homologous Sequence Analysis

A genome-wide synteny analysis was performed to compare the mitochondrial genome architectures of three *Pedicularis* species (*P. chinensis*, *P. kansuensis*, and *P. henryi*). Complete mitochondrial genome sequences of the three target species were acquired in FASTA format, followed by pairwise nucleotide alignments for all three genome combinations using BLASTN with a word size of 7 and an E-value cutoff of 1 × 10^−5^. Only homologous fragments with a minimum length of 500 bp and >80% sequence identity were retained from the alignment outputs, and the qualified fragments were further partitioned into direct and inverted syntenic blocks based on the relative strand orientation of the aligned sequences.

### 2.7. Phylogenetic Reconstruction

To determine the phylogenetic position of *P. henryi* within Lamiales, a maximum likelihood (ML) analysis was performed based on mitochondrial protein-coding genes (PCGs). A total of 18 mitochondrial genomes were retrieved from public databases. A set of conserved PCGs shared among all selected genomes was extracted, individually aligned using MAFFT (v7.427) (Table S1) [43], and trimmed with trimAl (v1.4) using the -gt 0.7 option [44]. The trimmed alignments were concatenated into a supermatrix for phylogenetic inference. The optimal substitution model (GTR + I + G) was selected with jModelTest (v2.1.10). ML analysis was conducted with RAxML (v8.2.10), and nodal support was assessed through 1000 bootstrap replicates [45].

## 3. Results

### 3.1. General Features of the Pedicularis henryi Mitochondrial Genome

**Figure 1:**
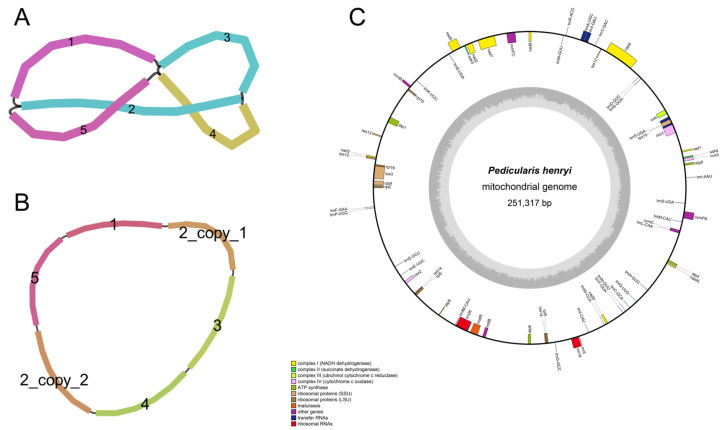
Mitochondrial genome assembly graph and resolved master circle of *P. henryi*. Note: (**A**) Bandage visualization of the draft assembly graph, with colored nodes representing assembled contigs and black lines indicating sequence overlaps between contigs. The labels ‘2_copy_1’ and ‘2_copy_2’ indicate the two copies of the long repeat region after splitting. (**B**) Resolved non-branched master circular structure after splitting the long repeat region into two independent copies to eliminate assembly branching. (**C**) Single circular map of the complete mitochondrial genome of *P. henryi*.

We de novo assembled and visualized the mitochondrial genome of *P. henryi*. The draft assembly showed a multi-branched closed structure with five contigs, where branching was driven by long repeat sequences (Figure 1A). We resolved this branching by splitting the repeat region into two independent copies, generating a final non-branched master circular mitochondrial genome (Figure 1B). The *P. henryi* mitochondrial genome yielded a single circular configuration with a total length of 251,317 bp and a GC content of 44.32% (Figure 1C, Table 1). Through comprehensive annotation, 63 unique genes were identified, comprising 36 protein-coding genes (PCGs), 24 transfer RNA genes, and three ribosomal RNA genes. The protein-coding regions extended over 9605 bp, representing approximately 3.82% of the complete genome. Among the PCGs, 14 were assigned to core functional categories associated with respiratory complexes, including ATP synthase, cytochrome c biogenesis, cytochrome c oxidase, ubiquinol cytochrome c reductase, and maturase. The remaining 22 PCGs fell into variable categories such as NADH dehydrogenase subunits, ribosomal proteins, a transporter membrane protein, and succinate dehydrogenase subunits. The tRNA gene complement consisted of 24 distinct species, with trnS-UGA present in three copies and trnN-GUU present in two copies, while all three rRNA genes (rrn5, rrn18, rrn26) existed as single copies.

### 3.2. Codon Usage Bias

**Figure 2:**
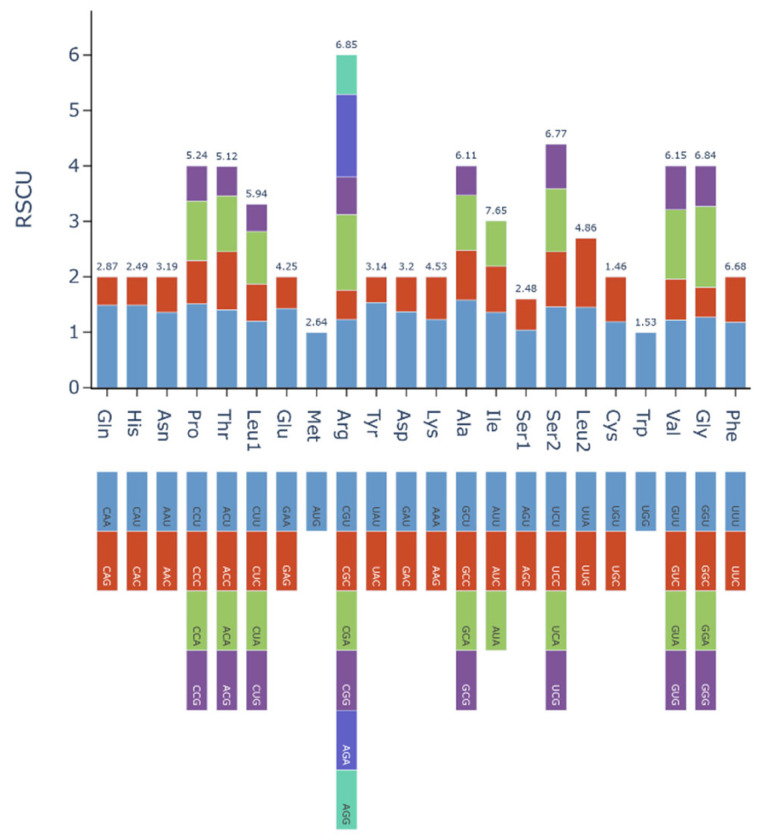
RSCU analysis of mitochondrial protein-coding genes in *P. henryi*.

Relative synonymous codon usage (RSCU) analysis of all mitochondrial PCGs in *P. henryi* demonstrated a pronounced preference for A/U-ending codons (≥90% of codons with RSCU > 1), with G/C-ending codons comprising nearly 93% of those with RSCU values below 1 (Figure 2). Notably, the initiation codon AUG (Met) and the sole tryptophan codon UGG both exhibited an RSCU value of 1, as these amino acids lack synonymous alternatives in the plant mitochondrial genetic code. Meanwhile, leucine (Leu), serine (Ser), and arginine (Arg) were the most frequently encoded amino acids in *P. henryi* mitochondrial PCGs, consistent with their having the highest number of synonymous codons among proteinogenic amino acids. The leucine-encoding UUA codon showed the highest RSCU value (7.65), followed by other strongly preferred codons such as GUU (Val, 6.85), CAU (His, 6.84), GCU (Ala, 6.68), GGU (Gly, 6.77), CAA (Gln, 6.11), and ACU (Thr, 5.94). These observations collectively confirm a pronounced, genome-wide preference for A/U-terminated codons in the *P. henryi* mitogenome.

### 3.3. Characterization of Repetitive Elements

**Figure 3:**
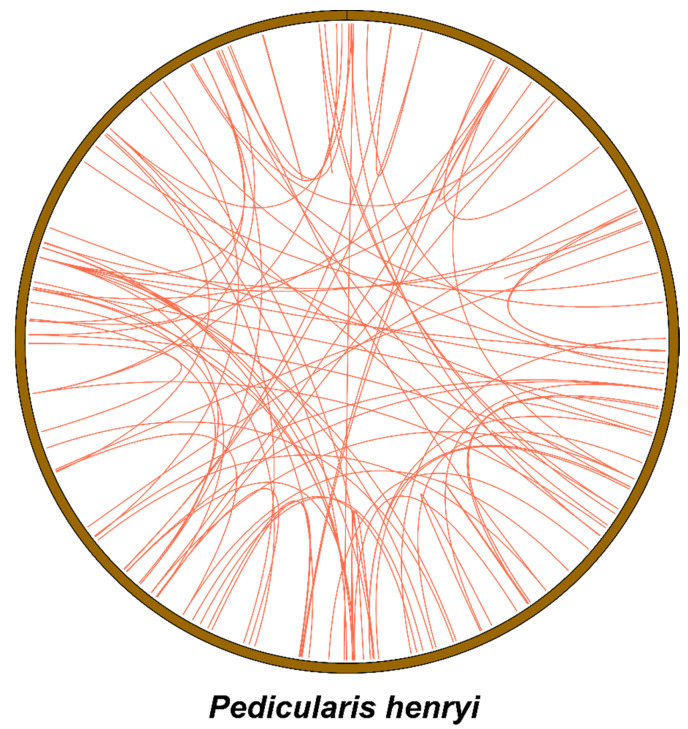
The distribution of interspersed repetitive elements in the mitochondrial genome of *P. henryi*. Note: The red lines inside the circle indicate the alignment links between homologous interspersed repeat sequences across the mitogenome.

Comprehensive characterization of repetitive elements in the *P. henryi* mitochondrial genome provides pivotal insights into its structural architecture, functional dynamics, and evolutionary processes. This study systematically profiled three major repeat classes, including simple sequence repeats (SSRs), tandem repeats, and interspersed repeats, to delineate their distribution and compositional features across the mitogenome (Figure 3). A total of 196 non-redundant repetitive elements were identified, consisting of 55 SSR loci, 7 tandem repeat arrays, and 134 interspersed repeats. Among SSRs, tetranucleotide motifs were the most abundant (43.6%, 24 loci), followed by mononucleotide (21.8%, 12 loci), dinucleotide (18.2%, 10 loci), trinucleotide (9.1%, 5 loci), and pentanucleotide (7.3%, 4 loci) motifs, with no A/T enrichment detected in mononucleotide repeats (Table S2). The 7 identified tandem repeats spanned 10–16 bp, with copy numbers ranging from 1.9 to 6.3 and sequence identity between repeat units of 72–100% (Table S3). The heterogeneity observed in these repeats suggests they have been subjected to differential selective constraints, and this sequence diversity may be linked to core biological processes such as mitogenome transcriptional regulation and the generation of genetic variation.

Interspersed repeats are major drivers of plant mitogenome evolution and are closely associated with genome size expansion, structural rearrangements, and sequence diversification. Of the 134 identified interspersed repeats, 76 direct and 58 palindromic repeats fell within the 0–100 bp interval, and repeat numbers decreased sharply as length increased. Only 12 direct and 9 palindromic repeats were detected in the 100–200 bp range, with even fewer in longer length bins. These elements exhibited substantial heterogeneity, with alignment lengths ranging from 31 to 5594 bp and sequence identity ranging from 74.0% to 100%. High-identity long repeats likely reside in conserved genomic regions with critical functional roles, while low-identity repeats may have accumulated mutations or been subjected to divergent selective pressures. Collectively, the classification and distribution of these repetitive elements provide a fundamental framework for investigating the evolutionary trajectory, rearrangement patterns, and adaptive evolution of *P. henryi* and its closely related taxa.

### 3.4. Prediction of RNA Editing Sites

**Figure 4:**
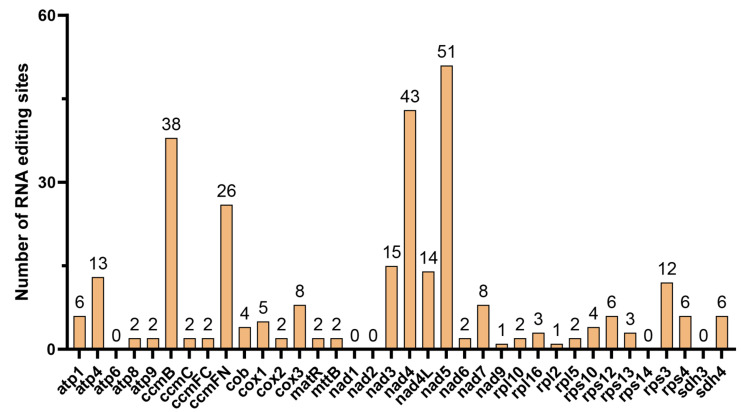
Number of C-to-U RNA editing sites in mitochondrial protein-coding genes of *P. henryi*.

Systematic identification and evaluation of C-to-U RNA editing events in the mitochondrial protein-coding genes (PCGs) of *P. henryi* revealed a total of 293 non-redundant editing sites, which were distributed across 31 of the 36 PCGs screened in this study (Figure 4). No RNA editing events were detected in 5 PCGs, namely atp6, nad1, nad2, rps14, and sdh3. Among all the genes analyzed, the nad5 gene harbored the highest number of editing sites with 51 loci, followed by the nad4 gene with 43 sites and the ccmB gene with 38 sites. When grouped by biological function, genes encoding NADH dehydrogenase subunits contributed the largest proportion of editing events, with a total of 134 sites detected across this gene family.

All predicted RNA editing events in this study were exclusively cytidine-to-uridine (C-to-U) base transitions. Analysis of codon position distribution showed that the majority of editing sites were located at the first and second bases of the codon, with only a small fraction of edits occurring at the third codon position. Simultaneous editing of both the first and second nucleotide positions within a single codon was observed in multiple genes, including *nad3*, *nad7*, *nad4*, *nad6*, *ccmFN*, *cob*, *nad5*, and *matR*. These dual-site edits predominantly resulted in the conversion of proline codons (CCT/CCC) to phenylalanine codons (TTT/TTC), driving non-synonymous changes in the encoded amino acid sequences.

### 3.5. Chloroplast-Derived Sequences in the Mitogenome

**Figure 5:**
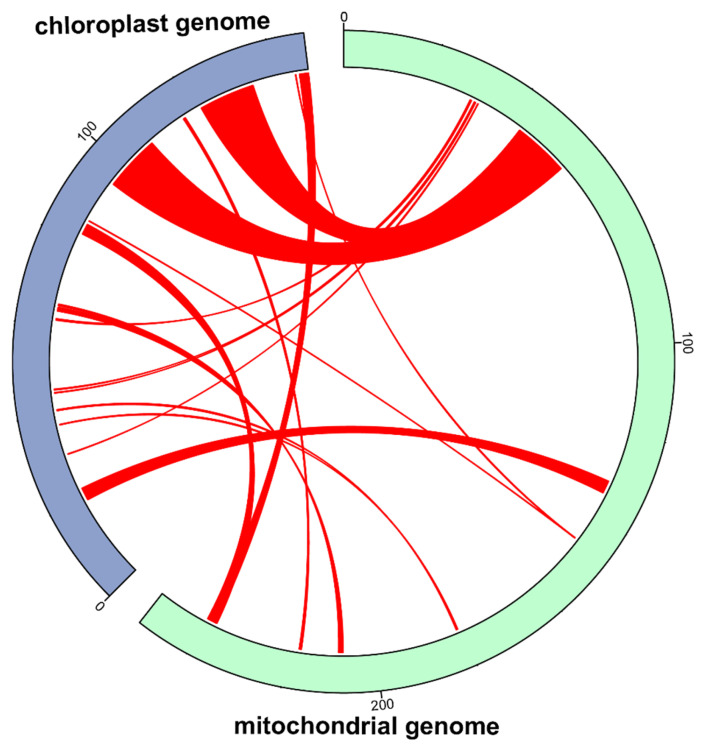
Mitochondrial genome map with MTPT fragment distribution. Note: Blue regions represent the chloroplast genome, light green regions represent the mitochondrial genome, while red ribbons indicate the 18 identified chloroplast-derived homologous DNA fragments in the mitochondrial genome.

Some chloroplast fragments were incorporated into the mitochondrial DNA throughout mitochondrial evolution, and the length of migrated fragments and sequence similarity varies among different species. Based on the sequence similarity analysis between the chloroplast and mitochondrial genomes, we identified 18 homologous DNA fragments of chloroplast origin in the mitochondrial genome of the studied species (Figure 5). The total insert length of these mitochondrial plastid DNAs (MTPTs) was 35,894 bp, accounting for approximately 15.0% of the 239,600 bp complete mitochondrial genome. Fragment 1 and fragment 2 are the longest transferred fragments, both with a length of 12,854 bp and were mapped to the same 42,953–55,806 bp region of the mitochondrial genome. Annotation of these homologous sequences revealed that these chloroplast-derived insertions covered both coding and non-coding regions of the chloroplast genome, including partial or complete protein-coding genes (PCGs), tRNA genes and intergenic spacer sequences.

### 3.6. Nucleotide Diversity Among Lamiales Mitochondrial Genes

**Figure 6:**
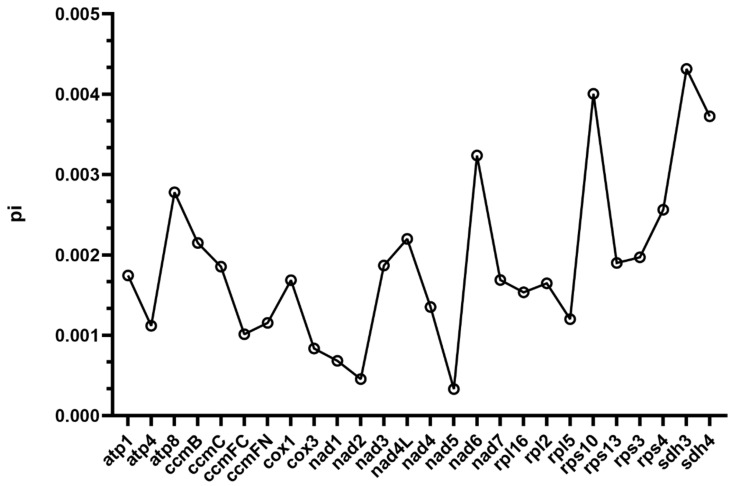
Nucleotide diversity analysis of mitochondrial protein-coding genes in *P. henryi*.

Estimation of nucleotide diversity (π) for mitochondrial protein-coding genes (PCGs) shared across *P. chinensis*, *P. kansuensis* and *P. henryi* yielded an overall average π value of 0.0018, with a broad range extending from 0.000331 in the NADH dehydrogenase gene nad5 to 0.004315 in the succinate dehydrogenase gene *sdh3* (Figure 6). Nearly half of the PCGs examined exhibited π values exceeding the overall average, pointing to considerable genetic variation within these coding regions, among which *sdh3*, *sdh4*, *rps10*, and *nad6* displayed the most prominent nucleotide diversity, all with π values above 0.0032. Genes associated with the succinate dehydrogenase complex, ATP synthase complex, and small ribosomal subunit proteins demonstrated notably higher sequence divergence, whereas genes encoding NADH dehydrogenase complex subunits and cytochrome c oxidase components generally showed strongly constrained variability with markedly low π values. These heterogeneous divergence patterns across different functional categories of mitochondrial genes suggest that distinct selective constraints and evolutionary regimes have operated on the mitogenome of *P. henryi*.

### 3.7. Mitochondrial Genome Synteny Analysis of Three Pedicularis Species

**Figure 7:**
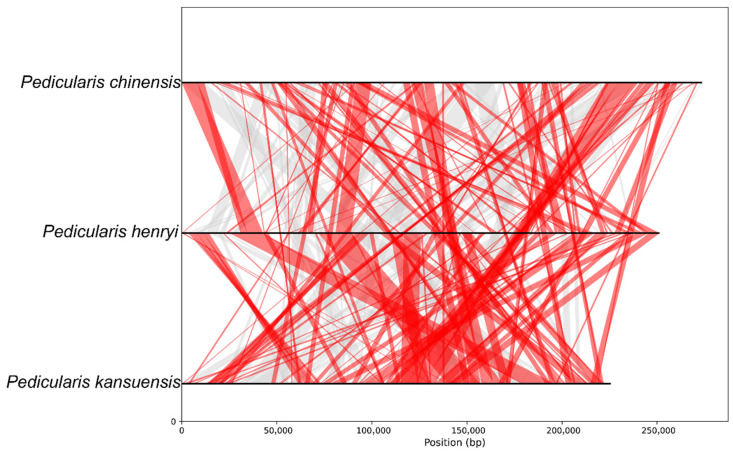
Mitochondrial genome synteny patterns among *P. chinensis*, *P. kansuensis* and *P. henryi*. Note: Gray regions represent collinear homologous segments aligned in the same strand, while red regions indicate inverted segments on the opposite strand.

The synteny analysis of Pedicularis mitochondrial genomes revealed distinct evolutionary patterns across three congeners, with *P. henryi* displaying unique structural characteristics. While *P. henryi* shared extensive high-identity homologous segments with *P. chinensis*, its mitochondrial genome was dominated by inverted regions (65.53% coverage) compared to only 22.01% same-orientation collinear regions, indicating profound structural rearrangement despite nucleotide-level conservation (Figure 7). In contrast, *P. henryi* showed balanced synteny with *P. kansuensis* (35.5% same-orientation, 34.36% inverted regions), whereas *P. chinensis* and *P. kansuensis* exhibited moderate rearrangement with 37.17% same-orientation collinearity and 40.15% inverted regions. These patterns confirm *P. henryi* as a genetically distinct lineage within Pedicularis, characterized by extreme mitochondrial genome restructuring relative to *P. chinensis* and more conserved architecture with *P. kansuensis*.

### 3.8. Phylogenetic Position of P. henryi

**Figure 8:**
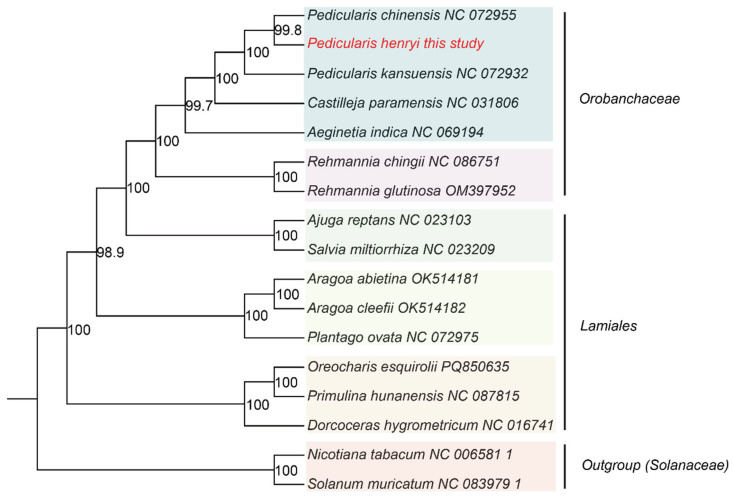
Phylogenetic tree of *P. henryi*. Note: Red text represents the mitochondrial sequence of *P. henryi* obtained in this study, while black text indicates the reference sequences downloaded from the NCBI database.

To elucidate the phylogenetic position of *P. henryi*, an ML tree was reconstructed based on concatenated nucleotide sequences of PCGs from 17 species, with two Solanaceae taxa (*Nicotiana tabacum* and *Solanum muricatum*) designated as outgroups. The resultant phylogeny yielded a well-resolved topology, with the majority of nodes receiving robust bootstrap support (≥98.9%) (Figure 8). Within the Lamiales clade, all sampled Orobanchaceae species formed a strongly supported monophyletic lineage (100% bootstrap support). *P. henryi* was initially placed as sister to *P. chinensis* with strong support (99.8%), and this subclade subsequently clustered with *P. kansuensis* (100% bootstrap support), constituting a well-supported monophyletic Pedicularis clade within the Orobanchaceae. The entire Orobanchaceae clade was positioned as sister to a large assemblage comprising representatives of Lamiaceae, Plantaginaceae, and Gesneriaceae with strong support (98.9% bootstrap support), consistent with the current understanding of Lamiales phylogeny.

## 4. Discussion

This study presents the first complete assembly and comprehensive characterization of the mitochondrial genome of *P. henryi*, a hemiparasitic species within Orobanchaceae. The assembled 251,317 bp circular mitogenome harbors a conserved core set of 36 protein-coding genes, 24 tRNAs and 3 rRNAs, with a low protein-coding proportion (3.82%) that aligns with the widespread evolutionary trajectory of plant mitogenomes shaped by non-coding region expansion [46]. Abundant repeat sequences within the genome mediate frequent homologous recombination, driving the dynamic structural complexity and alternative subgenomic conformations that are defining hallmarks of angiosperm mitochondrial genomes. Its genome size falls within the intermediate range of sequenced Orobanchaceae mitogenomes, highlighting the family’s striking size heterogeneity driven by lineage-specific repeat accumulation and intracellular gene transfer [47], with copy number variation of selected tRNA genes as a notable species-specific feature [48]. Bidirectional gene communication between mitochondria and the nucleus coordinates mitochondrial genome replication, expression, and maintenance, while mitochondrially encoded core oxidative phosphorylation subunits regulate stress responses and apoptosis, with stress-induced dysfunction triggering cytochrome c release to initiate the cell death cascade.

The strong A/U-ending codon preference in the *P. henryi* mitogenome is a conserved feature of plant mitochondrial genetic systems, shaped by the combined effects of mutational pressure and translational selection, with the distribution of non-preferred G/C-ending codons consistent with the AT-biased base composition of angiosperm mitogenomes [49]. Species-specific optimal codons with elevated RSCU values reflect translational selection matching cognate tRNA abundance [50], while the predominance of leucine, serine and arginine among encoded amino acids is a deeply conserved trait across plant mitogenomes [51].

Repetitive elements are the primary drivers of structural plasticity in the *P. henryi* mitogenome, with 196 non-redundant repeats identified as potential substrates for homologous recombination-mediated genome rearrangement. The predominance of tetranucleotide SSR motifs and lack of A/T enrichment in mononucleotide repeats are species-specific features deviating from the common repeat composition pattern in most angiosperms [52]. Heterogeneity in tandem repeat copy number and sequence identity suggests differential selective constraints during mitogenome evolution [53], while the length-dependent decrease in interspersed repeat abundance, along with their wide ranges of alignment length and sequence identity, indicates diverse evolutionary origins [54]. These abundant interspersed repeats are also the main driver of structural rearrangements detected in synteny analysis with congeneric species [55].

The exclusive detection of C-to-U RNA editing events confirms the deep conservation of this post-transcriptional modification mechanism in angiosperms. The 293 identified editing sites show marked gene-specific heterogeneity, which may reflect differential regulatory requirements for different mitochondrial genes. Most editing sites are concentrated at the first and second codon positions, leading to extensive non-synonymous substitutions, with dual-site editing in a subset of genes further modulating the hydrophobicity and secondary structure of encoded proteins [56]. Notably, the reduced number of RNA editing sites in *P. henryi* compared with non-parasitic Orobanchaceae species such as *Rehmannia chingii* is a distinctive feature, potentially associated with metabolic pathway simplification during parasitism evolution [57].

We identified 18 chloroplast-derived mitochondrial plastid DNA fragments in the *P. henryi* mitogenome, accounting for approximately 15.0% of the total genome length, revealing extensive intracellular gene transfer from chloroplasts to mitochondria. These fragments cover both coding and non-coding regions of the chloroplast genome, indicating large-scale integration of chloroplast DNA segments [58]. The colocalization of the two longest fragments in the same mitochondrial genomic region suggests that these insertions arose from a single or consecutive chloroplast DNA transfer event mediated by homologous recombination [59], the core molecular mechanism driving foreign DNA integration into the mitogenome, while reactive oxygen species-induced double-strand breaks in organelle genomes provide the primary trigger and raw material for this inter-organelle DNA transfer. The genomic proportion of chloroplast-derived insertions in *P. henryi* is comparable to the 13.14% ratio previously documented in the Stemona sessilifolia mitogenome [60], and markedly higher than the 7.68% contribution from predominantly short non-coding chloroplast-derived fragments in the Pontederia crassipes mitogenome [61]. Such variation in the scale and sequence composition of chloroplast-to-mitochondrion DNA transfer reflects lineage-specific evolutionary patterns of organelle genome exchange in angiosperms.

The average nucleotide diversity (π = 0.0018) of mitochondrial protein-coding genes across three *Pedicularis* species indicates high sequence conservation within the genus, consistent with the slow evolutionary rate typical of angiosperm mitogenomes. The wide range of π values and marked heterogeneity across functional gene families reflect differential selective constraints: core respiratory chain components are under strong purifying selection to maintain functional integrity [62], while genes encoding succinate dehydrogenase and small ribosomal subunit proteins show elevated nucleotide diversity, suggesting relaxed selective constraints [63].

Synteny analysis reveals high nucleotide sequence conservation coexisting with extensive structural rearrangement among *Pedicularis* mitogenomes [64]. *P. henryi* shares extensive high-identity homologous segments with *P. chinensis* but with predominantly inverted regions, indicating substantial genomic restructuring after species divergence, while its more balanced synteny with *P. kansuensis* suggests a closer evolutionary relationship in genomic architecture. This structural divergence is largely mediated by homologous recombination involving abundant repetitive elements. The maximum likelihood phylogenetic tree based on concatenated mitochondrial protein-coding genes confirms the monophyly of sampled Orobanchaceae species with robust bootstrap support, consistent with the APG IV taxonomic framework [65]. The clear clustering of *P. henryi* with its congeners clarifies intraspecific phylogenetic relationships within *Pedicularis*, and its topological congruence with chloroplast-based phylogenies supports coordinated evolution of the two organellar genomes [66].

## 5. Conclusions

This study reports the complete assembly and characterization of the *P. henryi* mitochondrial genome, revealing its structural organization, gene composition, codon usage bias, repetitive element distribution, RNA editing patterns, MTPT content, nucleotide diversity, syntenic relationships, and phylogenetic placement. The *P. henryi* mitogenome comprises a circular molecule of 251,317 bp with a conserved core gene set of 36 PCGs, 24 tRNAs, and three rRNAs, while exhibiting species-specific features including tRNA copy number variation, tetranucleotide-dominated SSRs, and an elevated proportion of MTPTs (15.0%). Repetitive elements drive extensive structural rearrangements that distinguish *P. henryi* from congeners despite high nucleotide sequence conservation. Differential selective constraints shape heterogeneous nucleotide diversity across mitochondrial genes, with core respiratory chain genes maintained under strong purifying selection and select functional genes experiencing relaxed selective pressure. Conserved mitochondrial PCGs effectively resolve the phylogenetic position of *P. henryi* and confirm the monophyly of Orobanchaceae, providing reliable molecular markers for phylogenetic studies within Lamiales.

## Figures and Tables

**Table 1:** Gene composition in the mitogenome of *P. henryi*.

Name of Genes	Gene List
ATP synthase	*atp8* (×2), *atp1* (×2), *atp9* (×2), *atp6* (×2), *atp4* (×2)
Cytochrome c oxidase	*cox3* (×2), *cox1* (×3), *cox2* (×2)
NADH dehydrogenase	*nad1* (×6), *nad5* (×4), *nad4* (×5), *nad6* (×2), *nad7* (×5), *nad 2* (×6), *nad3* (×2), *nad9* (×2), *nad4L* (×2)
Ubiquinol cytochrome c reductase	*cob* (×2)
Cytochrome c biogenesis	*ccmfc* (×3), *ccmb* (×2), *ccmc* (×2), *ccmfn* (×2)
Maturases	*matr* (×2)
Transport membrane protein	*mttb* (×2)
Succinate dehydrogenase	*sdh4* (×2), *sdh3* (×2)
Small subunit of ribosome	*rps10* (×3), *rps12* (×4), *rps13* (×2), *rps3* (×3), *rps4* (×2), *rps14* (×4)
Large subunit of ribosome	*rpl10* (×2), *rpl16* (×2), *rpl2* (×2), *rpl5* (×4)
tRNA	*trnI-AAU*, *trnS-UGA* (×3), *trnS-GGA*, *trnD-GUC*, *trnV-GAC*, *trnI-GAU*, *trnA-UGC*, *trnR-ACG*, *trnN-GUU* (×2), *trnK-UUU*, *trnF-GAA*, *trnP-UGG*, *trnS-GCU*, *trnE-UUC*, *trnfM-CAU*, *trnG-GCC*, *trnI-CAU*, *trnW-CCA*, *trnY-GUA*, *trnC-GCA*, *trnQ-UUG*, *trnH-GUG*, *trnL-CAA*, *trnM-CAU*
rRNA	*rrn26*, *rrn18*, *rrn5*

## Data Availability

The raw sequencing data generated in this study have been deposited in the GenBank database of the National Center for Biotechnology Information (NCBI) under the accession number PZ122839 (https://www.ncbi.nlm.nih.gov).

## Supplementary Materials

The following supporting information can be downloaded at: https://www.mdpi.com/article/10.3390/biology15070586/s1, Table S1: List of NCBI accession identifiers for mitochondrial genomes of species examined in this study; Table S2: Physical distribution of SSR loci within the mitochondrial genome of *P. henryi*; Table S3: Distribution of tandem repeats in the mitochondrial genome of *P. henryi*.

## References

[1] Friedman J.R., Nunnari J. (2014). Mitochondrial form and function. Nature.

[2] Nosek J., Tomáska L. (2003). Mitochondrial genome diversity: Evolution of the molecular architecture and replication strategy. Curr. Genet..

[3] Takenaka M., Verbitskiy D., Zehrmann A., Härtel B., Bayer-Császár E., Glass F., Brennicke A. (2014). RNA editing in plant mitochondria-connecting RNA target sequences and acting proteins. Mitochondrion.

[4] Mposhi A., Van der Wijst M.G., Faber K.N., Rots M.G. (2017). Regulation of mitochondrial gene expression, the epigenetic enigma. Front. Biosci..

[5] Smith D.R., Keeling P.J. (2015). Mitochondrial and plastid genome architecture: Reoccurring themes, but significant differences at the extremes. Proc. Natl. Acad. Sci. USA.

[6] Wang T., He H., Sun J., Man J., Li J., Sun C., Zhao Y., Kuang Z., Liu Y., Huang Z. (2025). From genome assembly to functional interpretation: An epistemological shift in understanding plant mitochondrial architecture. Front. Plant Sci..

[7] Skippington E., Barkman T.J., Rice D.W., Palmer J.D. (2015). Miniaturized mitogenome of the parasitic plant *Viscum scurruloideum* is extremely divergent and dynamic and has lost all nad genes. Proc. Natl. Acad. Sci. USA.

[8] Putintseva Y.A., Bondar E.I., Simonov E.P., Sharov V.V., Oreshkova N.V., Kuzmin D.A., Konstantinov Y.M., Shmakov V.N., Belkov V.I., Sadovsky M.G. (2020). Siberian larch (*Larix sibirica* Ledeb.) mitochondrial genome assembled using both short and long nucleotide sequence reads is currently the largest known mitogenome. BMC Genom..

[9] Gualberto J.M., Newton K.J. (2017). Plant mitochondrial genomes: Dynamics and mechanisms of mutation. Annu. Rev. Plant Biol..

[10] Ni Y., Li J., Tan Y., Shen G., Liu C. (2025). Advance in the assembly of the plant mitochondrial genomes using high-throughput DNA sequencing data of total cellular DNAs. Plant Biotechnol. J..

[11] Lai C., Wang J., Kan S., Zhang S., Li P., Reeve W.G., Wu Z., Zhang Y. (2022). Comparative analysis of mitochondrial genomes of *Broussonetia* spp. (Moraceae) reveals heterogeneity in structure, synteny, intercellular gene transfer, and RNA editing. Front. Plant Sci..

[12] Yu W.B., Liu M.L., Wang H., Mill R.R., Ree R.H., Yang J.-B., Li D.-Z. (2015). Towards a comprehensive phylogeny of the large temperate genus *Pedicularis* (Orobanchaceae), with an emphasis on species from the Himalaya-Hengduan Mountains. BMC Plant Biol..

[13] Yatoo M.I., Dimri U., Gopalakrishnan A., Karthik K., Gopi M., Khandia R., Saminathan M., Saxena A., Alagawany M., Farag M.R. (2017). Beneficial health applications and medicinal values of *Pedicularis* plants: A review. Biomed. Pharmacother..

[14] Liu R., Wang H., Yang J.B., Corlett R.T., Randle C.P., Li D.-Z., Yu W.-B. (2022). Cryptic Species Diversification of the *Pedicularis siphonantha* Complex (Orobanchaceae) in the Mountains of Southwest China Since the Pliocene. Front. Plant Sci..

[15] Tkach N., Ree R.H., Kuss P., Röser M., Hoffmann M.H. (2014). High mountain origin, phylogenetics, evolution, and niche conservatism of arctic lineages in the hemiparasitic genus *Pedicularis* (Orobanchaceae). Mol. Phylogenetics Evol..

[16] Li X., Yang J.B., Wang H., Song Y., Corlett R.T., Yao X., Li D.-Z., Yu W.-B. (2021). Plastid NDH Pseudogenization and Gene Loss in a Recently Derived Lineage from the Largest Hemiparasitic Plant Genus *Pedicularis* (Orobanchaceae). Plant Cell Physiol..

[17] Wang T., Li X., Tang C., Cao Z., He H., Ma X., Li Y., De K. (2024). Complete chloroplast genomes and phylogenetic relationships of *Pedicularis chinensis* and *Pedicularis kansuensis*. Sci. Rep..

[18] Wicke S., Naumann J., Müller K.F., Depamphilis C.W., Quandt D., Bellot S., Schneeweiss G.M. (2016). Mechanistic model of evolutionary rate variation en route to a nonphotosynthetic lifestyle in plants. Proc. Natl. Acad. Sci. USA.

[19] Li X., Lin C.Y., Yang J.B., Yu W.-B. (2020). De novo assembling a complete mitochondrial genome of *Pedicularis rex* (Orobanchaceae) using GetOrganelle toolkit. Mitochondrial DNA B Resour..

[20] Zervas A., Petersen G., Seberg O. (2019). Mitochondrial genome evolution in parasitic plants. BMC Evol. Biol..

[21] Zhang Q.Y., Chen Z., Sun H., Niu Y. (2023). Intraspecific floral colour variation in three *Pedicularis* species. Plant Divers..

[22] Zhang Q., Lu Z., Guo M., Kang J., Li J., He X., Wu J., Liu R., Dang J., Li Z. (2024). Responses of Three *Pedicularis* Species to Geological and Climatic Changes in the Qinling Mountains and Adjacent Areas in East Asia. Plants.

[23] Najer T., Doña J., Buček A., Sweet A.D., Sychra O., Johnson K.P. (2024). Mitochondrial genome fragmentation is correlated with increased rates of molecular evolution. PLoS Genet..

[24] Feng Y., Wicke S. (2025). Systemic organellar genome reconfiguration along the parasitic continuum in the broomrape family (Orobanchaceae). Plant Cell Physiol..

[25] Ichida H., Kazama T., Arimura S.I., Toriyama K. (2023). The mitochondrial and plastid genomes of *Oryza sativa* L. cv. Taichung 65. Plant Biotechnol..

[26] Murray M.G., Thompson W.F. (1980). Rapid isolation of high molecular weight plant DNA. Nucleic Acids Res..

[27] Li H. (2018). Minimap2: Pairwise alignment for nucleotide sequences. Bioinformatics.

[28] Koren S., Walenz B.P., Berlin K., Miller J.R., Bergman N.H., Phillippy A.M. (2017). Canu: Scalable and accurate long-read assembly via adaptive k-mer weighting and repeat separation. Genome Res..

[29] Langmead B., Salzberg S.L. (2012). Fast gapped-read alignment with Bowtie 2. Nat. Methods.

[30] Wick R.R., Judd L.M., Gorrie C.L., Holt K.E. (2017). Unicycler: Resolving bacterial genome assemblies from short and long sequencing reads. PLoS Comput. Biol..

[31] Wick R.R., Schultz M.B., Zobel J., Holt K.E. (2015). Bandage: Interactive visualization of de novo genome assemblies. Bioinformatics.

[32] Robinson J.T., Thorvaldsdóttir H., Winckler W., Guttman M., Lander E.S., Getz G., Mesirov J.P. (2011). Integrative genomics viewer. Nat. Biotechnol..

[33] Tillich M., Lehwark P., Pellizzer T., Ulbricht-Jones E.S., Fischer A., Bock R., Greiner S. (2017). GeSeq—Versatile and accurate annotation of organelle genomes. Nucleic Acids Res..

[34] Dunn N.A., Unni D.R., Diesh C., Munoz-Torres M., Harris N.L., Yao E., Rasche H., Holmes I.H., Elsik C.G., Lewis S. (2019). Apollo: Democratizing genome annotation. PLoS Comput. Biol..

[35] Chan P.P., Lowe T.M. (2019). tRNAscan-SE 2.0: Improved detection and functional classification of transfer RNA genes. Nucleic Acids Res..

[36] Greiner S., Lehwark P., Bock R. (2019). OrganellarGenomeDRAW (OGDRAW) version 1.3.1: Expanded toolkit for the graphical visualization of organellar genomes. Nucleic Acids Res..

[37] Edera A.A., Sanchez-Puerta M.V., Milone D.H. (2021). Deepred-Mt: Deep representation learning for predicting C-to-U RNA editing in plant mitochondria. Comput. Struct. Biotechnol. J..

[38] Beier S., Thiel T., Münch T., Scholz U., Mascher M. (2017). MISA-web: A web server for microsatellite prediction. Bioinformatics.

[39] Benson G. (1999). Tandem repeats finder: A program to analyze DNA sequences. Nucleic Acids Res..

[40] Krzywinski M., Schein J., Birol I., Connors J., Gascoyne R., Horsman D., Jones S.J., Marra M.A. (2009). Circos: An information aesthetic for comparative genomics. Genome Res..

[41] Chen C., Chen H., Zhang Y., Thomas H.R., Frank M.H., He Y.H., Xia R. (2020). TBtools: An integrative toolkit developed for interactive analyses of big biological data. Mol. Plant.

[42] Librado P., Rozas J. (2009). DnaSP v5: A software for comprehensive analysis of DNA polymorphism data. Bioinformatics.

[43] Katoh K., Standley D.M. (2013). MAFFT multiple sequence alignment software version 7: Improvements in performance and usability. Mol. Biol. Evol..

[44] Capella-Gutiérrez S., Silla-Martínez J.M., Gabaldón T. (2009). trimAl: A tool for automated alignment trimming in large-scale phylogenetic analyses. Bioinformatics.

[45] Stamatakis A. (2014). RAxML version 8: A tool for phylogenetic analysis and post-analysis of large phylogenies. Bioinformatics.

[46] Guo W., Grewe F., Fan W., Young G.J., Knoop V., Palmer J.D., Mower J.P. (2016). Ginkgo and Welwitschia Mitogenomes Reveal Extreme Contrasts in Gymnosperm Mitochondrial Evolution. Mol. Biol. Evol..

[47] Zhong Y., Yu R., Chen J., Liu Y., Zhou R. (2022). Highly active repeat-mediated recombination in the mitogenome of the holoparasitic plant *Aeginetia indica*. Front. Plant Sci..

[48] Zeng T., Ni Y., Li J., Chen H., Lu Q., Jiang M., Xu L., Liu C., Xiao P. (2024). Comprehensive analysis of the mitochondrial genome of *Rehmannia glutinosa*: Insights into repeat-mediated recombinations and RNA editing-induced stop codon acquisition. Front. Plant Sci..

[49] Shan Y., Li J., Duan X., Zhang X., Yu J. (2024). Elucidating the multichromosomal structure within the *Brasenia schreberi* mitochondrial genome through assembly and analysis. BMC Genom..

[50] Shen B., Shen A., Liu L., Tan Y., Li S., Tan Z. (2024). Assembly and comparative analysis of the complete multichromosomal mitochondrial genome of *Cymbidium ensifolium*, an orchid of high economic and ornamental value. BMC Plant Biol..

[51] Zhou P., Zhang Q., Li F., Huang J., Zhang M. (2023). Assembly and comparative analysis of the complete mitochondrial genome of *Ilex metabaptista* (Aquifoliaceae), a Chinese endemic species with a narrow distribution. BMC Plant Biol..

[52] Zhou S., Wei N., Jost M., Wanke S., Rees M., Liu Y., Zhou R. (2023). The Mitochondrial Genome of the Holoparasitic Plant *Thonningia sanguinea* Provides Insights into the Evolution of the Multichromosomal Structure. Genome Biol. Evol..

[53] Qu K., Chen Y., Liu D., Guo H., Xu T., Jing Q., Ge L., Shu X., Xin X., Xie X. (2024). Comprehensive analysis of the complete mitochondrial genome of *Lilium tsingtauense* reveals a novel multichromosome structure. Plant Cell Rep..

[54] Szandar K., Krawczyk K., Myszczyński K., Ślipiko M., Sawicki J., Szczecińska M. (2022). Breaking the limits—Multichromosomal structure of an early eudicot *Pulsatilla patens* mitogenome reveals extensive RNA-editing, longest repeats and chloroplast derived regions among sequenced land plant mitogenomes. BMC Plant Biol..

[55] Qu K., Liu D., Sun L., Li M., Xia T., Sun W., Xia Y. (2024). De novo assembly and comprehensive analysis of the mitochondrial genome of *Taxus wallichiana* reveals different repeats mediate recombination to generate multiple conformations. Genomics.

[56] Kan S.L., Shen T.T., Gong P., Ran J.-H., Wang X.-Q. (2020). The complete mitochondrial genome of *Taxus cuspidata* (Taxaceae): Eight protein-coding genes have transferred to the nuclear genome. BMC Evol. Biol..

[57] Han Y., Feng Y.L., Wang J., Zhu S.-S., Jin X.-J., Wu Z.-Q., Zhang Y.-H. (2024). Comprehensive Analysis of the Complete Mitochondrial Genome of *Rehmannia chingii*: An Autotrophic Species in the Orobanchaceae Family. Genes.

[58] Dong S., Zhao C., Chen F., Liu Y., Zhang S., Wu H., Zhang L., Liu Y. (2018). The complete mitochondrial genome of the early flowering plant *Nymphaea colorata* is highly repetitive with low recombination. BMC Genom..

[59] Wang X., Li L.L., Xiao Y., Chen X.-Y., Chen J.-H., Hu X.-S. (2021). A complete sequence of mitochondrial genome of *Neolamarckia cadamba* and its use for systematic analysis. Sci. Rep..

[60] Xie Y., Liu W., Guo L., Zhang X. (2024). Mitochondrial genome complexity in *Stemona sessilifolia*: Nanopore sequencing reveals chloroplast gene transfer and DNA rearrangements. Front. Genet..

[61] Hao Z., Jiang X., Pan L., Guo J., Chen Y., Li J., Liu B., Guo A., Luo L., Jia R. (2024). The complete mitochondrial genome of *Pontederia crassipes*: Using HiFi reads to investigate genome recombination and gene transfer from chloroplast genome. Front. Plant Sci..

[62] Li L., Fu H., Altaf M.A., Wang Z., Lu X. (2024). The complete mitochondrial genome assembly of *Capsicum pubescens* reveals key evolutionary characteristics of mitochondrial genes of two *Capsicum* subspecies. BMC Genom..

[63] Chen H., Huang L., Yu J., Miao Y., Liu C. (2023). Mitochondrial genome of *Artemisia argyi* L. suggested conserved mitochondrial protein-coding genes among genera *Artemisia*, *Tanacetum* and *Chrysanthemum*. Gene.

[64] Li Y., Chen S., Zhang F., Xia M. (2026). Structural characterization of the plastid genome and comparative mitochondrial genomics of *Eupatorium lindleyanum* (Asteraceae): Evolutionary dynamics and phylogenetic insights. BMC Plant Biol..

[65] Xia Z., Wen J., Gao Z. (2019). Does the Enigmatic Wightia Belong to Paulowniaceae (Lamiales). Front. Plant Sci..

[66] Wang M., Zhang S., Zhang L. (2024). The complete chloroplast genome sequences of three *Pedicularis* species (Orobanchaceae). Genet. Mol. Biol..

